# The Use of Tobacco

**Published:** 1872-10

**Authors:** 


### THE USE OF TOBACCO.

The injury arising from the use of tobacco is to a great extent negative, although, as a deleterious agent, we will see tobacco exerting sometimes very positive influences.

It would be curious, if it were not pitiful, to fully know what sublime schemes, what profound thoughts, what plans—wide as humanity and deep as the foundations of civilization—have melted away and vanished through the dreamy imbecility of tobacco smoke. Yet when the enervating influence of the pleasing siren has passed off, the young and the ambitious, or, it may be, the old and wise, start once more into activity and seem about to realize, in actual fact, their cherished and noble ideals, when once again, and yet again, forever, the soothing influence of tobacco beguiles and deludes, like a silent dream, every faculty of action and every impulse of energy. Thus postponement and procrastination and tobacco dim the brightest intellects, and weaken the pulsations of the noblest hearts, until suddenly, all at once, old age is here. The years of brightness and promise and youth are passed and gone, and the "night when no man can work" has come.

Tobacco seems to act principally upon the organic nervous system, depressing all its functions as the nutrition of the body, the circulation of blood, and even the reproductive powers. The influence of tobacco upon the action of the heart, when it is used in undue quantity for a long period of time, is very peculiar, and is well deserving of notice. The heart displays, in time, more or less irregularity in its action. This is evinced in palpitation, in tumultuous motion, of that organ, especially when lying upon the left side, confining the space of its action.—There may be a loud and thumping sound of pulsation. There is sometimes a gurgling or squirting sensation in the region of the heart.—Not unfrequently there is very prominent, upon the slightest mental excitement, a distinct sound about the heart, as of rubbing leather, that is, a creaking sound with every heart beat, like rubbing upon glass with the finger. These symptoms are attended at times with a sense of vertigo or dizziness, and a person so affected often feels that it is safest to sit down a little while to prevent his staggering or even falling. The eyes sometimes feel as though they were fixed in the head immovably, and perhaps crossed, and the person affected wonders in his own mind if he does not present a singular appearance to beholders.

All this time the pulse varies. It is always feeble, frequently rapid, "a nervous pulse," but very often, indeed, irregular and greatly intermittent. Sometimes it is very rapid for a few seconds, then slow and labored; now a heavy throb, again a few hurried, thread-like, and very faint pulsations, scarcely, indeed, perceptible, and anon, a stop altogether, until the patient, frightened and greatly concerned—if he is at all attentive to his pulse—lets go the wrist lest his increasing anxiety culminate in his dropping dead. Sometimes he may fall dying. This is a form of heart disease entirely owing to the abuse of tobacco.

In the attacks of prostration, to which allusion is made, the heart is possibly, after many years of conflict with an insidious poison, finally subdued. It is almost powerless while the brain as a consequence suffers from a deficiency of blood. The face is deadly pale. The end of the nose is cold and pinched. The patient sighs feebly and gasps for breath. He sinks, wonderfully limp and white, close to the bed. A gulping effort to vomit, frequently unsuccessful from sheer prostration, is followed by a state of unconsciousness resembling death. This condition of *syncope* is worse than ordinary fainting. Where the presence of nicotine, or tobacco poison, is universal throughout the system, there is much to contend with in addition to the state of simple fainting. This prostration, sometimes fatal, tells a long story, and to many a plain one, of the slow entrance, but at length universal prevalence of the poisonous principle of tobacco—nicotine—in the system.

It is plain that here is a condition that it will take a considerable time to rectify. To ever recover so far that a recurrence of the fearful symptoms above described shall not be at any and every season impending, implies a complete revolution and reproduction throughout the whole structure. Long time, suitable remedies, proper nutriment, and an entire abandonment of the use of tobacco will—assisted by the process of interstitial substitution of healthy substance for poisoned atoms—accomplish the desired object. Until that point is clearly attained—and it will require from one to three years to effect the complete "casting out" of the evil spirit—it will be found that every cigar smoked will threaten, in some degree, to bring on an attack of syncope. It is surprising to see how *fearful*, so to speak, the system has now become of tobacco, although it seemed once to be absolutely impregnable to its assaults.

